# Integrative hospital treatment in older patients to benchmark and improve outcome and length of stay – the *In-HospiTOOL* study

**DOI:** 10.1186/s12913-019-4045-x

**Published:** 2019-04-23

**Authors:** Alexander Kutz, Daniel Koch, Antoinette Conca, Ciril Baechli, Sebastian Haubitz, Katharina Regez, Ursula Schild, Zeljka Caldara, Fahim Ebrahimi, Stefano Bassetti, Jens Eckstein, Juerg Beer, Michael Egloff, Vladimir Kaplan, Tobias Ehmann, Claus Hoess, Heinz Schaad, Ulrich Wagner, Sabina de Geest, Philipp Schuetz, Beat Mueller

**Affiliations:** 1 Medical University Department, Division of General Internal and Emergency Medicine, Kantonsspital Aarau, Tellstrasse, CH-5001 Aarau, Switzerland; 2 Division of Internal Medicine, University Hospital Basel, Basel, Switzerland; 3 Internal Medicine Department, Kantonsspital Baden, Baden, Switzerland; 4 Internal Medicine Department, Kreisspital Muri, Muri, Switzerland; 5 Internal Medicine Department, Spital Zofingen, Zofingen, Switzerland; 6 Internal Medicine Department, Kantonsspital Muensterlingen, Muensterlingen, Switzerland; 7 Internal Medicine Department, Spital Interlaken, FMI, Interlaken, Switzerland; 8 Swiss Federal Office for Statistics, Neuchâtel, Switzerland; 9 Department of Public Health of the Faculty of Medicine, University Hospital Basel, Basel, Switzerland

**Keywords:** Health services research, Integrated care, Interprofessional, Polymorbidity, Transition, Discharge planning, Clinical outcomes, Length of hospital stay, Readmission, Resource allocation

## Abstract

**Background:**

A comprehensive in-hospital patient management with reasonable and economic resource allocation is arguably the major challenge of health-care systems worldwide, especially in elderly, frail, and polymorbid patients. The need for patient management tools to improve the transition process and allocation of health care resources in routine clinical care particularly for the inpatient setting is obvious. To address these issues, a large prospective trial is warranted.

**Methods:**

The “Integrative Hospital Treatment in Older patients to benchmark and improve Outcome and Length of stay” (In-HospiTOOL) study is an investigator-initiated, multicenter effectiveness trial to compare the effects of a novel in-hospital management tool on length of hospital stay, readmission rate, quality of care, and other clinical outcomes using a time-series model. The study aims to include approximately 35`000 polymorbid medical patients over an 18-month period, divided in an observation, implementation, and intervention phase. Detailed data on treatment and outcome of polymorbid medical patients during the in-hospital stay and after 30 days will be gathered to investigate differences in resource use, inter-professional collaborations and to establish representative benchmarking data to promote measurement and display of quality of care data across seven Swiss hospitals. The trial will inform whether the “In-HospiTOOL” optimizes inter-professional collaboration and thereby reduces length of hospital stay without harming subjective and objective patient-oriented outcome markers.

**Discussion:**

Many of the current quality-mirroring tools do not reflect the real need and use of resources, especially in polymorbid and elderly patients. In addition, a validated tool for optimization of patient transition and discharge processes is still missing. The proposed multicenter effectiveness trial has potential to improve interprofessional collaboration and optimizes resource allocation from hospital admission to discharge. The results will enable inter-hospital comparison of transition processes and accomplish a benchmarking for inpatient care quality.

**Electronic supplementary material:**

The online version of this article (10.1186/s12913-019-4045-x) contains supplementary material, which is available to authorized users.

## Background

One of the major challenges of health-care systems, governments, and societies worldwide is a comprehensive in-hospital patient management with reasonable and economic resource allocation [1], especially in frail, polymorbid, and elderly patients. A lower level of education and a cognitive impairment are additional risk factors. [2]. Novel and optimized diagnostic and therapeutic approaches have increased expectation of life, resulting in increased polymorbidity and complexity. Moreover, elderly patients with high demands for medical, but also nursing and social care call for a tighter allocation of our health care resources [3, 4]. Whereas emergency hospitalizations are facilitated by chronic disease burden, co-morbidities, and frailty, the trigger for hospital admission is often a per se minor acute event (e.g. urinary tract infection), disrupting the fragile bio-psycho-social homeostasis of polymorbidity. In many cases, a post-acute transfer to a nursing care facility is required. The need for patient management tools to improve the transition process and allocation of health care resources in routine clinical care particularly for the inpatient setting is obvious.

### Challenges and current evidence

Misutilization and suboptimal resource allocation challenges safe and efficient, patient-centered in-hospital transition from the emergency department (ED) to a medical ward, and transition to home or post-acute care facilities [5]. Errors that lead to unplanned readmissions and preventable deaths are more common in polymorbid patients [6]. Since the majority of medical inpatients with chronic illnesses become hospitalized via ED (non-electively), optimized resource use must start at the ED with an improved triage.

The optimal organization of routine medical ward care in mostly polymorbid, elderly medical patients received less alertness than the handling of specific diseases. Specifically, there is a lack of large trials focusing on polymorbid patients and their objective outcomes [7]. The inter-professional team care approach with a comprehensive geriatric in-hospital assessment has been found effective to increase patients’ likelihood of being alive and living in their own homes after an emergency admission to hospital [8]. Conversely, many earlier studies were unable to link interdisciplinary team care interventions to affect existing metrics, partly because of limited methodology and outcome measures [9]. As recently shown in a review article, several innovative interprofessional and interdisciplinary healthcare interventions on medical wards usually have chosen length of hospital stay, mortality rates, readmission, or functionality as their primary outcome measures [10]. However, most interventions have not shown any effect on these patient-oriented outcomes and are therefore debatable and inconsistent. Few evidence suggests that an improved interprofessional collaboration would reduce adverse effects of care. Thus, a significant reduction in length of hospital stay have been reported due to contemporaneous secular reasons [11], which most of the published interventions did not reduce additionally. The majority of inpatient setting interventions were unlikely to reduce readmissions, neither to reduce mortality rates, nor to diminish need for nursing facilities after discharge. However, generalizability of these findings remains weak due to discrepancies between different healthcare systems and heterogeneous (inter-)national standards of care.

Recently, innovative strategies to synergize the concepts of implementation science, precision medicine, and learning health care systems have been advocated [12]. Using this experience, we will integrate evidence-based strategies (e.g., training, supervision, quality monitoring tools, system change interventions) into real-world practice [13].

### Rationale of the study and overall aim

The main reason for the large controversy of the different tool’s effectiveness in elderly, polymorbid patients is mainly explained by the current lack of evidence. Hence, a large prospective multicenter trial is warranted to investigate the effects of a patient in-hospital management tool (“In-HospiTOOL”). Using an interrupted time series (ITS) model to gather detailed treatment and outcome data of elderly, polymorbid medical patients during the in-hospital stay and 30 days after admission, we will investigate differences in resource use (Module 1), interprofessional collaborations (Module 2), and establish a representative benchmarking database to promote measurement and display of quality of care [14] across different sized Swiss hospitals (Module 3).

## Methods/design

### Aim and study design

The “In-HospiTOOL” study is a quasi-experimental investigator-initiated, multicenter effectiveness trial investigating the effects of a new patient in-hospital management tool to improve length of hospital stay and other outcomes using an ITS model. The overall 18-month study time is divided in a 6-month observational phase, a 6-month implementation phase, and a 6-month intervention phase.

### Setting, study sites and characteristics of participants

The multicenter trial includes seven secondary and tertiary care hospitals within Northern Switzerland. This allows to collect representative nation-wide patient-oriented data from polymorbid patients. All senior executive leaders have reassured full support for an optimal implementation of the “In-HospiTOOL” in their hospitals. Upon hospital admission, consecutive adult (age ≥ 18 years), polymorbid (> 1 diagnose) medical inpatients will be included in our study. Except for non-medical and non-adult patients there will be no exclusion criteria.

### Patient population

#### Intervention population

To reflect “daily practice”, we include consecutive adult medical inpatients independent of their diagnosis during the observation, implementation, and intervention period, respectively, into the analysis - like an intention-to-treat approach.

#### Control population

For our statistical approach as outlined below, we request data from the Federal Statistical Office to provide a nationwide comparability. We will use data on length of hospital stay. Age, gender, health care insurance, place of residence, main diagnosis, comorbidities, and study center will be used for statistical adjustment.

### Data collection process

The study period is divided into three 6-month phases, whereas the observation and the intervention phase until the end of January 2019 are season-matched, interrupted by a run-in non-season-matched implementation phase. The period of “In-HospiTOOL” implementation in the participating hospitals will be devoted to technical implementation, training of involved study personnel, nurses, social workers, physicians, and pilot testing. We will collect data throughout all three study periods by using electronic medical records and will contact all patients 30 days after hospital admission by phone interview. Data from the structured phone interviews will be stored on a data base using the secuTrial© software (secuTrial®; interActive Systems GmbH, Berlin, Germany).

### Theoretical aspects, hypothesis

#### Module 1 resource (mis-)utilization & allocation

**Fig. 1:**
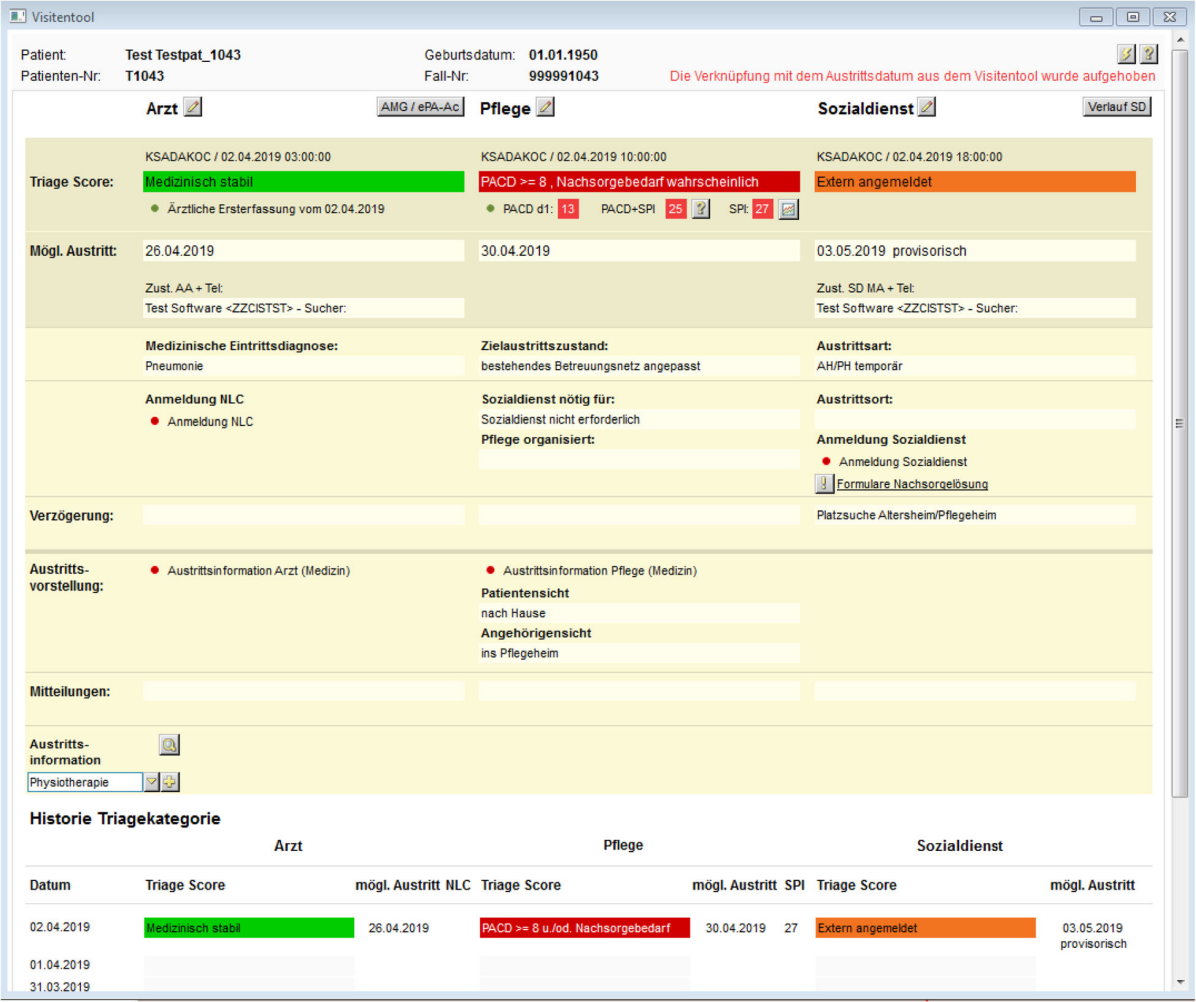
The “*Visitentool*” (german for “ward-round tool”). Inter-professional collaboration via an electronical communication platform. Nursing and physician staff as well as social services daily assess the clinical and functional situation about possible discharge (using simple, intuitive color coding) and propose possible discharge dates. Also, reasons for delays in discharge are being monitored

We hypothesize that implementing the “In-HospiTOOL” in a nationwide multicenter setting will significantly shorten length of hospital stay without compromising patient outcomes and functional independence. Systematic early determination of clinical stability, estimation of the post-acute care referral probability (using the Post-acute care discharge [PACD] score [15]), fixing a possible discharge date after initial ED assessment as well as a tight interprofessional collaboration enabled through an electronical communication platform (“Visitentool”, Fig. 1) will result in decreased waiting times contributing to the shortening of length of hospital stay [16–18].

#### Module 2 Interprofessional collaboration, integral hospital and post-acute patient transition

**Fig. 2:**
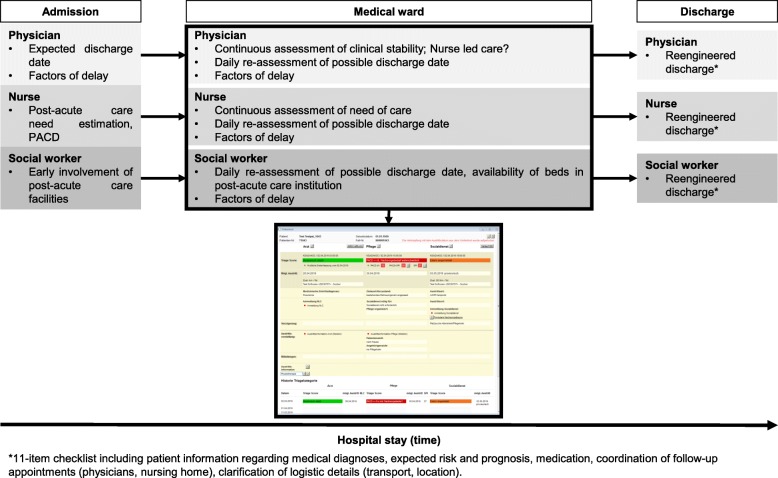
The “In-HospiTOOL”. An integrative patient management tool. The “In-HospiTOOL” has three components involving admission (inter-professional initial assessment, *“Ersterfassung”*), medical ward (inter-professional daily re-assessment, *“Visitentool”*), and discharge (inter-professional patient education, reengineered discharge [32]). PACD, Post-Acute Care Discharge [15, 18]

Transparent and systematic interprofessional communication will reveal factors for delay in these polymorbid patients (pending diagnostics, medical treatments, administrative and organizational elements) throughout the hospital stay. Doing so, we will identify regional and socioeconomic (e.g., health care insurance status) differences in the patient care continuum. We hypothesize that longitudinal observation of patient transition will further allow measurement of effective time from initial request to a post-acute care institution to effective transfer with corresponding internal and external delaying factors (Fig. 2). We will systematically examine patient satisfaction, hypothesizing that it will not decrease upon the study intervention. Implementing a reengineered discharge questionnaire including the teach-back methodology, we aim to improve patient education [19, 20]. This effort will not only strengthen interprofessional coordination and communication, but also increase health care continuity across all hospital transition steps. Moreover, we systematically investigate reasons for low satisfaction.

#### Module 3 benchmarking to advice health care authorities and stakeholders

The buildup of a large dataset including comprehensive patient information (demographics, clinical, organizational, health insurance status) will be a basis for future data sharing in Switzerland [21]. We hypothesize that this dataset from several hospitals will allow to identify associations of management factors and outcome data, thereby facilitating a better understanding how interventions affect patient outcomes. Convocation of a multi-professional sounding board with tailored implementation interventions [22] will be inevitable to built-up a data warehouse and thus, broad dissemination of our results with potential to improve health care service also in non-participating health care institutions.

### Endpoints

All patients will be daily assessed during hospitalization and contacted 30 days after admission by phone for a structured and blinded interview to assess primary and secondary endpoints.

The primary endpoint of this study is length of hospital stay within 30 days after admission including readmissions during this period (corresponding to Module 1). Length of hospital stay will be verified based on hospital data for the index hospital stay and complemented by 30-day interviews regarding possible secondary hospitalizations.

Secondary endpoints (corresponding to Module 1–3) include measures of patient-oriented outcomes:

- in-hospital and all-cause 30-day mortality
- unplanned readmissions or unplanned general practitioner / ED visits
- delaying factors of ED- and medical ward’s flow
- effective time to hospital discharge after involving external institutions (time from transfer application to transfer)
- institutionalization
- satisfaction with ED-, ward-, and discharge process
- functional status (incl. Quality of life) using the EuroQol Group 5-Dimension Self-Report Questionnaire (see Additional file 1) [23].

To study hospital internal processes and effects of interprofessionality, we will look at compliance and agreement of the three health professions (physicians, nursing, and social workers) in the use of “In-HospiTOOL”, and delays from the anticipated to the effective discharge date as compared to discharge date anticipated by the different health care professionals on admission and during the course of the hospital stay. We will use the above-mentioned outcome data set as benchmark to establish a risk-adjusted resource and quality cockpit to compare different hospitals and demographics (corresponding to Module 3).

### Independent variables

The primary exposure variable of interest is the intervention, i.e. the implementation of the “In-HospiTOOL”. As outlined in the statistical plan, we will adjust our model to the following covariates: demographics (age, gender, health care insurance, home of residence [home versus facility]), main diagnosis (grouped using the “International Classification of Diseases (ICD-10)” [24]), comorbidities (using the Elixhauser comorbidity index [19]), and study center.

### Implementation strategies and components

#### Electronical tools

Based on a sounding board discussion, we designed and optimized the different electronical study tools in collaboration with the technical providers. In addition, we defined and implemented a structured data export, first, to guarantee user’s compliance measurement during study time, second, to enable adoption of tool items during the implementation period based on preliminary analyses, and, third, to establish a patient-oriented data “cockpit” for benchmarking.

#### User-oriented key data benchmarking

Based on the sounding board consensus, we designed a structured questionnaire (see Additional file 2) to perform phone interviews with all included patients 30 days after hospital admission to assess their outcome data and satisfaction. Each study site establishes a local interview team performing the 30-day structured follow-up interviews. Depending on the cumulative number of patients, between 3 and 7 part-time working persons were recruited by each study site, respectively, and were finally instructed by the core study nurse team.

#### Coaching

Before starting the implementation phase, a minimum of two interprofessional onsite visits at each study site ensures full information and compliance of the local staff (physicians, nurses, social workers, therapeutic disciplines) about participating in the In-HospiTOOL study and preparing them how to use the newly designed electronical study tools in the later implementation phase. Before and during the intervention phase, a minimum of two interprofessional onsite visits at each study site will be repeated to guarantee an optimal monitoring quality and a high usability. To guarantee a standardized staff education, we designed (in cooperation with the “Berner Bildungszentrum Pflege”) and provide a teaching video to all study sites, where the appropriate usage of the electronical study tools has been described comprehensively (https://youtu.be/bNyRPucs-FQ). To finally guarantee a maximal utilization and a correct application in daily clinical routine, we encourage all the study sites to introduce a local supervisor. The local supervisor will monitor compliance of the In-HospiTOOL usage, such as the physician’s initial assessment, PACD score, Visitentool (on weekdays), reengineered discharge form, and intervenes if appropriate. The required profiles of personnel qualification are assertiveness and well-experienced clinical nurses, nursing experts with clinical background, or case managers.

### Statistical analysis plan and sample size

The primary analysis population includes all consecutively recruited patients following an intention-to treat (ITT) principle. The number of patients lost to follow-up will be minimized by every effort. The per-protocol (PP) population analysis will be prospectively defined to exclude patients in whom major protocol violations have occurred. Specifically, the following criteria will lead to exclusion from the PP population: major violation of study inclusion or study exclusion criteria, patients with missing information from the In-HospiTOOL forms, or missing follow-up interviews.

We will compare all endpoints between all three study phases in the overall ITT population, the PP population and within predefined subgroups as discussed below. We will analyze all endpoints in an adjusted manner for the main covariates such as age, gender, health care insurance, home of residence, main diagnosis, comorbidities, and study center. Regarding sample size considerations, we include consecutive patients in each hospital over a 6-month period for the observation, implementation, and the intervention period, respectively. Given the large number of patients per study site seen in routine (i.e. between 2`500 and 8`000 polymorbid medical inpatients) per year, we estimate to enroll approximately ~ 35′000 patients over 18 months of recruitment. This large amount of patient data will provide adequate power to investigate the effect of introducing a patient care tool in the overall medical hospitalized patient population and allow for subgroup analyses, as well as important post-hoc analyses.

We will perform two complementary, quasi-experimental analyses to estimate the effect of the intervention: difference in differences and ITS.

#### Difference in differences

To quantify an overall effect on length of hospital stay after implementing the “In-HospiTOOL”, we fit a patient-level multivariate linear regression model. It will include lengths of hospital stay of “control” hospitals, all risk adjusters listed above in the manuscript, and a time variable for study weeks. The dependent variable will be length of hospital stay (days in hospital). We will test for interaction between the intervention period and the intervention population and will assess whether there is a difference in the change (slope) in length of hospital stay over time between the two study populations. The intervention and control groups will have different baseline characteristics, however both groups will be exposed to similar alterations in outcomes over time without the interventional program.

#### Interrupted time series (ITS) model

We will analyze the trends in length of hospital stay from start of observation through the end of the intervention period (18 months). For this purpose, we will conduct an ITS as a sensitivity analysis. We will implement the ITS using generalized estimating equations (GEE), to examine linear trends in weekly, hospital-level, risk-adjusted length of hospital stays. ITS models will be tested for autocorrelation to control for seasonal trends using the Durbin-Watson statistics [25]. We will analyze the change in trend between all three time periods. This approach allows us to distinguish between a possible effect of the intervention and a difference from underlying trends facing both study groups and can also help to analyse whether the intervention effects will sustain over time. We will calculate weekly-adjusted length of hospital stay using linear GEE that includes all lengths of hospital stay from the control and intervention populations and all above mentioned risk factors. We will graph weekly length of hospital stay for all populations over time and use the estimated weekly means for control and intervention patients to assess for a weekly difference between the two groups. Finally, we explore whether the adjusted weekly length of hospital stay decreases in the intervention period and whether there will be an independent time trend effect induced by the intervention only [26].

To summarize, we will use four statistically hypothesis tests during each period: (i) Are there significant trends in length of hospital stay change during the period. (ii) Will the trend differ between the control and intervention populations (the interaction between time and control or intervention conditions) during the period. (iii) Will the trend during the intervention period differ from the trend during the observation period within all three conditions. (iiii) Will the magnitude of the altered trend between the intervention and the observation period differ between the three conditions (the interaction between the change in slope and intervention or control conditions). These models will also be implemented for the analyses of the secondary endpoints.

## Discussion

### Scientific significance

Performing clinical trials in the usual care setting (“comprehensive effectiveness health services research”) has the potential to identify and demonstrate relevant patient-oriented outcomes to the institutions where they are performed and at the same time to yield information that may be generalizable to the health care system at large [27].

Health care spending in Switzerland is among the highest worldwide and has been rising due to a change in demographics. Still, a high scientific evidence regarding performance, safety, and cost-effectiveness of specific integrative multi-professional care models tailored to the Swiss health care system is largely lacking. The “In-HospiTOOL” is an electronic integrative multi-professional inpatient management tool to better understand the variegated health care processes. Using a standardized but also personalized procedure, the In-HospiTOOL will optimize the interprofessional management of polymorbid acutely-ill patients from ED admission to hospital discharge. Herewith, we will improve transparency, resource use, patient outcomes, patient satisfaction, and functional status. An overall cost-effectiveness analysis will be performed separately as a secondary analysis. We anticipate that the results of the In-HospiTOOL-study will contribute to improved (inter-)national inpatient health care and reduce overall costs.

In addition to the main interventional trial, gathering of data from around 35`000 polymorbid patients from seven Swiss hospitals will help to establish a nationwide framework involving important stakeholders of the Swiss health care system. Interprofessional and interdisciplinary collaboration are prerequisites for an improved sustainable patient-oriented health care delivery with an optimal resource allocation. This will lead to a more efficient patient transition with decreased risk for hospital associated adverse outcomes. Besides, the large dataset will allow the comparison of different outcomes of different patient populations across different hospitals with each individual health care strategies. In addition, health insurances and policy authorities might profit from these data to conceptualize new reimbursement strategies in the polymorbid inpatient setting.

Such integrated comparative effectiveness health services research relies on the collaboration of care providers and health care systems as active partners in defining the objectives of the research rather than as passive consumers of its product [27]. This pragmatic study will hopefully enforce a change in “culture” by rethinking and redefining traditional regulatory and ethical standards (e.g. patient informed consent and overall engagement in research) in this paradigm of low risk research [28].

As the evidence about interventions for optimization of in-hospital patient transition is scarce, some hospitals have designed their own tools. However, performance, cost- effectiveness, safety, transferability, and external validity of these interventions are poorly investigated [29, 30]. Different health care stakeholders lack scientific evidence to support and authorize changes or to improve transition of polymorbid patients. Thus, a further validation of interprofessional interventions and quality benchmarks is urgently needed to close this gap in current health care discussions. Patient management, transition and length of hospital stay without negatively affecting patient outcomes and functional independence despite polymorbidity should be of highest relevance in this real-world and pragmatic multicenter setting [10, 31].

Our study has several potential limitations. First, inclusion of about 35`000 patients over the 18-month study time frame is ambitious. However, based on our large experience from previous multicenter studies, a well-established multicenter research network, and the high prevalence of hospitalized multimorbid patients eligible for this trial, we are convinced that the trial is feasible. The second limitation of this trial is the lack of randomization. Methodologically, in a trial whose intervention focuses on the process of care, neither patient-level randomization nor a randomized cross-over design was feasible as a carry-over effect would have biased the control group. For a cluster randomized trial, a large number of similar clusters (i.e. hospitals) would be needed – which is challenging in Switzerland particularly in light of the limited study budget. Therefore, we chose an ITS model in a quasi-experimental study design using administrative data from non-participating hospitals as a control group. Doing so, we account for differences in the patient population that may occur due to epidemiological variations. Third, as a pragmatic trial with a bundled intervention, it will be challenging to understand which part of our intervention shows clinical effects, leading to potential participation bias. Nonetheless, the large amount of health care data collected will also allow to provide scientific evidence as to which elements of patient management are influenced by the intervention.

In conclusion, this pragmatic comparative effectiveness health services research trial will inform whether the implementation of a novel electronic tool for inpatient management optimizes inter-professional collaboration and thereby reduces length of hospital stay without derogating subjective and objective patient-oriented measures. Our trial will help to compare between diverse transition processes within seven different hospitals and to create a benchmarking for inpatient care quality. This study synergizes national networks and, thus, has the potential to become a cornerstone in the present public healthcare discussion.

## Additional files

Additional file 1: EuroQol Group 5-Dimension Self-Report Questionnaire. (PDF 149 kb)

Additional file 2: Structured questionnaire to perform phone interviews with all included patients 30 days after hospital admission to assess their outcome data and satisfaction. (PDF 265 kb)

## Abbreviations

CTU: Clinical Trial Unit
ED: Emergency department
GEE: Generalized estimating eqs.
ICD: International Classification of Diseases
In-HospiTOOL: Integrative Hospital Treatment in Older patients to benchmark and improve Outcome and Length of stay
IRB: Institutional review board
ITS: Interrupted time series
ITT: Intention-to treat
PACD: Post-acute care discharge
PP: Per-protocol
