# Supplementary material for: Integrative hospital treatment in older patients to benchmark and improve outcome and length of stay – the In-HospiTOOL study
Source: BMC Health Serv Res. 2019 Apr 23;19:237. doi: 10.1186/s12913-019-4045-x (PMC6480877; doi:10.1186/s12913-019-4045-x)
Supplement: Supplementary file 1 — EuroQol Group 5-Dimension Self-Report Questionnaire. (PDF 149 kb) [file 12913_2019_4045_MOESM1_ESM.pdf]

EQ-5D-5L

Health Questionnaire

English version

SCRIPT FOR TELEPHONE INTERVIEW

GENERAL INTRODUCTION

It is suggested that the telephone interviewer follows the script of the EQ-5D. Although allowance should be made for the interviewer's particular style of speaking, the wording of the questionnaire instructions should be followed as closely as possible. In the case of the EQ-5D descriptive system on pages 2 and 3, the precise wording must be followed.

It is recommended that the interviewer has a copy of the EQ-5D in front of him or her as it is administered over the telephone. This enables the respondent's answers to be entered directly on the EQ-5D by the interviewer on behalf of the respondent (i.e. the appropriate boxes on pages 2 and 3 are marked and the scale on page 4 is marked at the point indicating the respondent's 'health today'). The respondent should also have a copy of the EQ-5D in front of him or her for reference. If the respondent asks for clarification, the interviewer can help by re-reading the question verbatim. The interviewer should not try to offer his or her own explanation but suggest that the respondent uses his or her own interpretation.

If the respondent has difficulty regarding which box to mark, the interviewer should repeat the question verbatim and ask the respondent to answer in a way that most closely resembles his or her thoughts about his or her health today.

© 2012 EuroQol Research Foundation. EQ-5D™ is a trade mark of the EuroQol Research Foundation. UK (English) v1.3

INTRODUCTION TO EQ-5D

(Note to interviewer: please read the following to the respondent)

We are trying to find out what you think about your health. I will first ask you some simple questions about your health TODAY. I will then ask you to rate your health on a measuring scale. I will explain what to do as I go along but please interrupt me if you do not understand something or if things are not clear to you. Please also remember that there are no right or wrong answers. We are interested here only in your personal view.

EQ-5D DESCRIPTIVE SYSTEM: INTRODUCTION

First I am going to read out some questions. Each question has a choice of five answers. Please tell me which answer best describes your health TODAY.

Do not choose more than one answer in each group of questions.

(Note to interviewer: it may be necessary to remind the respondent regularly that the timeframe is TODAY. It may also be necessary to repeat the questions verbatim)

EQ-5D DESCRIPTIVE SYSTEM

MOBILITY

First I'd like to ask you about mobility. Would you say that:

1. **You have no problems in walking about?**
2. **You have slight problems in walking about?**
3. **You have moderate problems in walking about?**
4. **You have severe problems in walking about?**
5. **You are unable to walk about?**

(Note to interviewer: mark the appropriate box on the EQ-5D questionnaire)

SELF-CARE

Next I'd like to ask you about self-care. Would you say that:

1. **You have no problems washing or dressing yourself?**
2. **You have slight problems washing or dressing yourself?**
3. **You have moderate problems washing or dressing yourself?**
4. **You have severe problems washing or dressing yourself?**
5. **You are unable to wash or dress yourself?**

(Note to interviewer: mark the appropriate box on the EQ-5D questionnaire)

© 2012 EuroQol Research Foundation. EQ-5D™ is a trade mark of the EuroQol Research Foundation. UK (English) v1.3

USUAL ACTIVITIES

Next I'd like to ask you about usual activities, for example work, study, housework, family or leisure activities. Would you say that:

1. **You have no problems doing your usual activities?**
2. **You have slight problems doing your usual activities?**
3. **You have moderate problems doing your usual activities?**
4. **You have severe problems doing your usual activities?**
5. **You are unable to do your usual activities?**

(Note to interviewer: mark the appropriate box on the EQ-5D questionnaire)

PAIN / DISCOMFORT

Next I'd like to ask you about pain or discomfort. Would you say that:

1. **You have no pain or discomfort?**
2. **You have slight pain or discomfort?**
3. **You have moderate pain or discomfort?**
4. **You have severe pain or discomfort?**
5. **You have extreme pain or discomfort?**

(Note to interviewer: mark the appropriate box on the EQ-5D questionnaire)

ANXIETY / DEPRESSION

Finally I'd like to ask you about anxiety or depression. Would you say that:

1. **You are not anxious or depressed?**
2. **You are slightly anxious or depressed?**
3. **You are moderately anxious or depressed?**
4. **You are severely anxious or depressed?**
5. **You are extremely anxious or depressed?**

(Note to interviewer: mark the appropriate box on the EQ-5D questionnaire)

© 2012 EuroQol Research Foundation. EQ-5D™ is a trade mark of the EuroQol Research Foundation. UK (English) v1.3

EQ VAS: INTRODUCTION

(Note to interviewer: if possible, it might be useful to send a visual aid (i.e. the EQ VAS) before the telephone call so that the respondent can have this in front of him or her when completing the task)

Now, I would like to ask you to say how good or bad your health is TODAY.

I'd like you to try to picture in your mind a scale that looks a bit like a thermometer. Can you do that? The best health you can imagine is marked 100 (one hundred) at the top of the scale and the worst health you can imagine is marked 0 (zero) at the bottom.

EQ VAS: TASK

I would now like you to tell me the point on this scale where you would put your health today.

(Note to interviewer: mark the scale at the point indicating the respondent's 'health today'. Now, please write the number you marked on the scale in the box below)

THE RESPONDENT'S HEALTH TODAY = ☐

Thank you for taking the time to answer these questions.

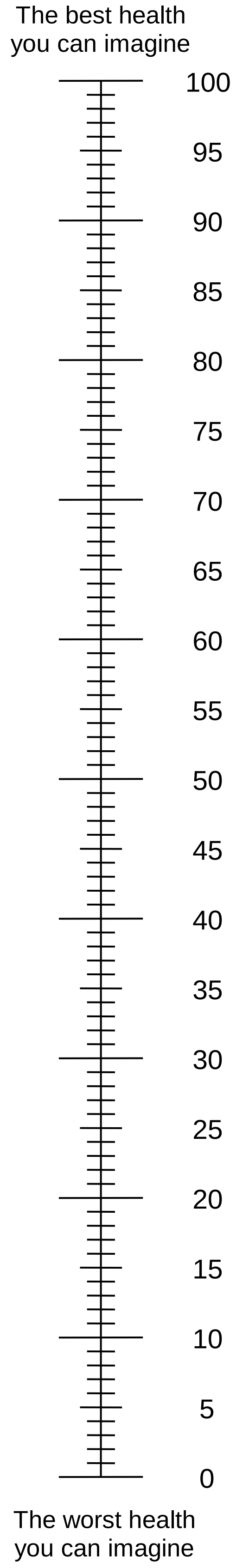

© 2012 EuroQol Research Foundation. EQ-5D™ is a trade mark of the EuroQol Research Foundation. UK (English) v1.3
