# Supplementary material for: Integrative hospital treatment in older patients to benchmark and improve outcome and length of stay – the In-HospiTOOL study
Source: BMC Health Serv Res. 2019 Apr 23;19:237. doi: 10.1186/s12913-019-4045-x (PMC6480877; doi:10.1186/s12913-019-4045-x)
Supplement: Supplementary file 2 — Structured questionnaire to perform phone interviews with all included patients 30 days after hospital admission to assess their outcome data and satisfaction. (PDF 265 kb) [file 12913_2019_4045_MOESM2_ESM.pdf]

In-HospiTOOL – 30-day interview

2.1 After you left the hospital: Did you ever have to go back to a hospital by emergency/unplanned/unexpectedly?

2.2 After you left the hospital: Did you ever have to visit an emergency department by emergency/unplanned/unexpectedly?

2.3 After you left the hospital: Did you ever have to visit a general practitioner by emergency/unplanned/unexpectedly?

3.1 How satisfied were you with the stay in the emergency department 30 days ago on a scale from 0 -10?

3.2 How satisfied were you with the hospital stay 30 days ago in general, on a scale from 0 -10?

3.2.1 What did you appreciate most during your stay?

3.2.2 What did you appreciate least during your stay?

4.0 How did you feel about the duration of your hospitalization?
- too long - ideally - too short - cannot be recorded

4.1 Did you feel well informed about your diagnosis and treatment? on a scale from 0 -10? - unknown

4.5 Did you get the opportunity to ask questions?
- always - mostly - sometimes - rather not - never - I had no questions - cannot be recorded

4.6 Have you received clear answers to your questions?
- always - mostly - sometimes - rather not - never - I had no questions - can’t be recorded

4.7 Have you been explained the purpose of the medication you are taking at home?
- yes, entirely; - mostly - partially - rather not - no, not at all
- I didn’t have to take medication at home - can’t be recorded

4.8 How do you rate the quality of treatment (by physicians and nurses)?
- excellent - very good - good - less well - bad - cannot be recorded

4.2 Have you been involved in planning your discharge?

4.3 Have you been asked to repeat the given information by the nursing- and physician staff in your own words?

4.4 Was everything well prepared for your life at home respectively your post-hospitalization care (e.g. medications, assistive equipment)?

4.9 How was the organization of your hospital discharge?
- excellent - very good - good - not so good - poor - cannot be recorded

5. *Current situation = today, 30 days after hospital admission*
Before hospitalization (period: four weeks) = within the four weeks before the hospital admission

-> Please tick the one from the following statements which better describes your health today.

5.1.1 -5.1.5

MOBILITY
I have no problems in walking about ✓
I have slight problems in walking about ❑
I have moderate problems in walking about ❑
I have severe problems in walking about ❑
I am unable to walk about ❑

SELF-CARE
I have no problems washing or dressing myself ❑
I have slight problems washing or dressing myself ✓
I have moderate problems washing or dressing myself ❑
I have severe problems washing or dressing myself ❑
I am unable to wash or dress myself ❑

USUAL ACTIVITIES *(e.g. work, study, housework, family or leisure activities)*
I have no problems doing my usual activities ❑
I have slight problems doing my usual activities ❑
I have moderate problems doing my usual activities ✓
I have severe problems doing my usual activities ❑
I am unable to do my usual activities ❑

PAIN / DISCOMFORT
I have no pain or discomfort ❑
I have slight pain or discomfort ❑
I have moderate pain or discomfort ❑
I have severe pain or discomfort ✓
I have extreme pain or discomfort ❑

ANXIETY / DEPRESSION
I am not anxious or depressed ❑
I am slightly anxious or depressed ❑
I am moderately anxious or depressed ❑
I am severely anxious or depressed ❑
I am extremely anxious or depressed ✓

5.1.6

- We would like to know how good or bad your health is TODAY.
- This scale is numbered from 0 to 100.
- 100 means the best health you can imagine.
  0 means the worst health you can imagine.

5.2.1-5.2.5

-> Please tick the one from the following statements which better describes your health within <u>four weeks before hospital admission.</u>

**MOBILITY**	
I have no problems in walking about	✓
I have slight problems in walking about	☐
I have moderate problems in walking about	☐
I have severe problems in walking about	☐
I am unable to walk about	☐
**SELF-CARE**	
I have no problems washing or dressing myself	☐
I have slight problems washing or dressing myself	✓
I have moderate problems washing or dressing myself	☐
I have severe problems washing or dressing myself	☐
I am unable to wash or dress myself	☐
**USUAL ACTIVITIES** *(e.g. work, study, housework, family or leisure activities)*	
I have no problems doing my usual activities	☐
I have slight problems doing my usual activities	☐
I have moderate problems doing my usual activities	✓
I have severe problems doing my usual activities	☐
I am unable to do my usual activities	☐
**PAIN / DISCOMFORT**	
I have no pain or discomfort	☐
I have slight pain or discomfort	☐
I have moderate pain or discomfort	☐
I have severe pain or discomfort	✓
I have extreme pain or discomfort	☐
**ANXIETY / DEPRESSION**	
I am not anxious or depressed	☐
I am slightly anxious or depressed	☐
I am moderately anxious or depressed	☐
I am severely anxious or depressed	☐
I am extremely anxious or depressed	✓

5.2.6

- We would like to know how good or bad your health is TODAY.
- This scale is numbered from 0 to 100.
- 100 means the <u>best</u> health you can imagine.
  0 means the <u>worst</u> health you can imagine.

We would like to know how good or bad your health was before your hospital admission (within 4 weeks).

5.3 Today, do you need assistance for:

- drug intake/provision?
- eating/drinking?
- excretion (urinating and passing stools)?

5.4 Four weeks before hospital admission, did you need assistance for:

- drug intake/provision?

- eating/drinking?
- excretion (urinating and passing stools)?

6.1 Has your housing situation changed since your hospital stay?

If yes:
- New, retirement home/elderly care home/nursing home
- Still in hospital
- Rehospitalization (back in hospital)
- New, living with a family member/carer
- Still in acute geriatrics, transitional care
- New, assisted living
- New, hospice/palliative care
- Still in rehabilitation
- Still in short-term care or respite care (aka “Ferienbett”)
- others

6.2 Care situation today: Do you need any assistance in activities of daily living?

If yes, who is providing this assistance?
- Institution (Rehabilitation, hospital, nursing home etc.)
- Spitex/home-care (nursing care or equivalent)
- Spitex/home-care (domestic work or equivalent)
- Family/reference person
- Home meal service ("meals-on-wheels")
- Transport service
- others

7. Now here are some questions about your health status:

7.1 Do you have problems with urinary incontinence (loss of bladder control)?

If yes:
- newly occurred
- less than before hospital admission (four weeks)
- more than before hospital admission (four weeks)
- unchanged
- unknown

If no:
- No problem before hospital admission (four weeks)
- Already a problem before hospital admission (four weeks)
- unknown

7.2 Do you have problems with bowel incontinence (inability to control bowel movements)?

If yes:
- newly occurred
- less than before hospital admission (four weeks)
- more than before hospital admission (four weeks)
- unchanged

- unknown

If no:
- No problem before hospital admission (four weeks)
- Already a problem before hospital admission (four weeks)
- unknown

7.3 Do you suffer from diarrhea?

If yes:
- newly occurred
- less than before hospital admission (four weeks)
- more than before hospital admission (four weeks)
- unchanged
- unknown

If no:
- No problem before hospital admission (four weeks)
- Already a problem before hospital admission (four weeks)
- unknown

7.4 Have you lost weight since hospital discharge?

7.5 How is your appetite on a scale from 0 – 10?

7.5.1
- less than before hospital admission (four weeks)
- more than before hospital admission (four weeks)
- same
- unknown

7.6
Have you fallen since hospital discharge?
If yes: 7.6.1 How often? once several times unknown

7.6.2 Have you had a bone fracture?

7.6.3
Have you fallen before hospital discharge?
If yes: 7.6.4 How often? once several times unknown

7.6.5 Have you had a bone fracture?

7.7 Are you currently suffering from sleep disorder (problems with falling asleep/sleep trough the night)?

7.7.1
If yes:
- newly occurred
- less than before hospital admission (four weeks)
- more than before hospital admission (four weeks)

- unchanged
- unknown

If no:

- No problem before hospital admission (four weeks)
- Already a problem before hospital admission (four weeks)
- unknown

7.8 Are you currently suffering from a strong, unusual fatigue?

7.8.1

If yes:

- newly occurred
- less than before hospital admission (four weeks)
- more than before hospital admission (four weeks)
- unchanged
- unknown

If no:

- No problem before hospital admission (four weeks)
- Already a problem before hospital admission (four weeks)
- unknown

7.9 Do you have diabetes?

7.9.1

If yes: Are your daily blood sugar levels now under better control than before hospitalization?

- Better
- Unchanged
- Worse
- unknown

7.10 Do you currently suffer from shortness of breath?

7.10.1 Classification according to NYHA

I = no symptoms and no limitation in ordinary physical activity, e.g. no shortness of breath when walking, climbing stairs etc.
II= Mild symptoms (mild shortness of breath and/or angina) and slight limitation during ordinary activity.
III= Marked limitation in activity due to symptoms, even during less-than-ordinary activity, e.g. walking short distances (20–100 m). Comfortable only at rest.
IV= Severe limitations. Experiences symptoms even while *at rest*. Mostly bedbound.

7.10.2 Did you suffer from shortness of breath before hospitalization (four weeks)?

I = no symptoms and no limitation in ordinary physical activity, e.g. no shortness of breath when walking, climbing stairs etc.
II= Mild symptoms (mild shortness of breath and/or angina) and slight limitation during ordinary activity.
III= Marked limitation in activity due to symptoms, even during less-than-ordinary activity, e.g. walking short distances (20–100 m). Comfortable only at rest.

IV= Severe limitations. Experiences symptoms even while *at rest*. Mostly bedbound.

7.11 Do you smoke? (all types of tobacco consumption/with or without lung inhalation)

7.11.1 Current amount (compared to before hosp.)?

- more
- unchanged
- less
- unknown

7.11.2 Did anybody in hospital discussed smoking cessation?

7.12 Did you have a bleeding since hospital discharge (such as nosebleed, hematoma, blood in your stools or sputum)?

8.1 Are you currently employed?

- No, even not before hospitalization
- No, I have not been working since hospitalization
- Yes
    - Workload?
    - Since when do you work again?

- unknown
